# Tailored Thienopyridine Therapy: No Urgency for CYP2C19 Genotyping

**DOI:** 10.1161/JAHA.112.000131

**Published:** 2013-04-24

**Authors:** Pierre Fontana, Marco Cattaneo, Christophe Combescure, Jean‐Luc Reny

**Affiliations:** 1 Division of Angiology and Haemostasis, Geneva University Hospital and Faculty of Medicine (Geneva Platelet Group), Thonex, Switzerland (P.F.); 2 Medicina 3, Ospedale San Paolo, Dipartimento di Scienze della Salute, Università degli Studi di Milano, Milan, Italy (M.C.); 3 Division of Clinical Epidemiology, Geneva University Hospital and Faculty of Medicine, Switzerland (C.C.); 4 Division of Internal Medicine, and Rehabilitation, Trois‐Chêne Hospital, Geneva University Hospital and Faculty of Medicine (Geneva Platelet Group), Switzerland (J.L.R.)

**Keywords:** pharmacogenetics, platelet, thienopyridine

## Abstract

Between 20% and 50% of cardiovascular patients treated with clopidogrel, an anti‐P2Y12 drug, display high on‐treatment platelet reactivity (HTPR) and are not adequately protected from major adverse cardiovascular events (MACE). Despite a minor influence of the CYP2C19*2 genetic variant on the pharmacodynamic response to clopidogrel (5% to 12%) and a limited or absent value for predicting stent thrombosis and MACE, this latter polymorphism is currently considered an important candidate to tailor anti‐P2Y12 therapy during percutaneous coronary intervention. Seven studies have examined the value of CYP2C19*2 for predicting HTPR in comparison to a specific pharmacodynamic assay (VASP assay). Overall, the summarized sensitivity of the CYP2C19*2 genotype for predicting HTPR was 37.6% (95% CI: 32.2 to 43.3%), yielding a negative likelihood ratio of only 0.77 (95% CI: 0.68 to 0.86) which confirms its limited value as a routine clinical aid. A tailored anti‐P2Y12 treatment strategy restricted to CYP2C19*2 carriers may be of some help, but this restrictive approach leaves out noncarriers with HTPR. As for platelet function testing, there is currently no convincing data to support that using CYP2C19*2 genotyping as a tailored anti‐P2Y12 treatment would be an effective strategy and there is no urgency for CYP2C19 genotyping in clinical practice. Strategies incorporating genotyping, phenotyping, and clinical data in a stratified and sequential approach may be more promising.

## Introduction

Clopidogrel exerts its antithrombotic effect through irreversible inhibition of the platelet receptor for adenosine diphosphate (ADP) P2Y12. Between about 20% and 50% of patients treated with clopidogrel display high on‐treatment platelet reactivity (HTPR)^1^ and are not adequately protected from MACE. In the era of personalized medicine, effective strategies are needed to identify these patients and thus to tailor their antiplatelet treatment.

As HTPR on clopidogrel seems to be strongly heritable (h^2^=0.73),^2^ genotyping could theoretically help to identify patients at risk. Clopidogrel is a prodrug that needs to be metabolized to its active metabolite by cytochrome P450 (CYP) isoforms in the liver. Various loss‐ and gain‐of‐function genotypes of CYP isoforms are known to affect the response to clopidogrel. In particular, CYP2C19 loss‐of‐function variant *2 (rs4244285) has been linked both to a poor pharmacodynamic response to clopidogrel and to an increased risk of recurrent cardiovascular events, best evidenced in patients treated with percutaneous coronary interventions (PCI) and for the outcome of stent thrombosis.^3–4^ However, recent metanalyses have challenged this link between CYP2C19*2 and MACE.^5–7^ The reported association between loss‐of‐function alleles and poor cardiovascular outcomes was found to suffer from bias due to small‐study effects,^6–7^ with no risk increase being found in a pooled analysis of studies involving more than 500 patients.^8^ These inconsistencies in the observed relation between CYP2C19*2 and MACE are likely explained by the fact that CYP2C19*2 has only a minor influence (5% to 12%) on the pharmacodynamic response to clopidogrel.^2,9–11^

**Figure 1.**
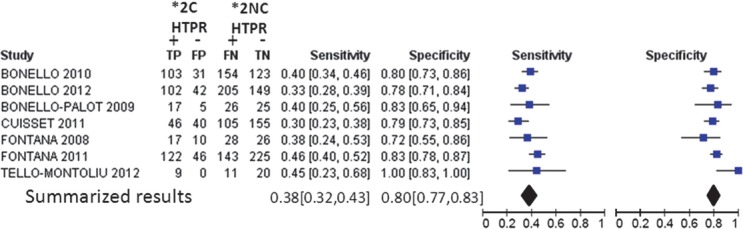
Sensitivity and specificity of the 2C19*1/*2 polymorphism for detecting high on‐treatment platelet reactivity (HTPR), as based on the vasodilator‐stimulated phosphoprotein (VASP) assay performed in clopidogrel‐treated patients. Patients are classified as either 2C19*2 carriers (*2C), corresponding to carriers of 1 or 2 *2 alleles, or 2C19*2 noncarriers (*2NC), corresponding to *1 homozygotes. The global sensitivity and specificity are depicted as a black diamonds. TP indicates true positives; FP, false positives; FN, false negatives; TN, true negatives.

The capacity of CYP2C19*1/*2 genotyping to predict HTPR has been examined in several studies using various platelet function tests, including VASP assay, which is highly specific for P2Y12 receptor inhibition.^12^ In a PubMed search conducted on October 25, 2012 using the terms “clopidogrel,” “vasodilator‐stimulated phosphoprotein,” and “cytochrome,” we identified 22 studies, 7 of which provided substantive data on the association between CYP2C19 genotypes and HTPR.^10,13–18^ As shown in the Figure1, the summarized sensitivity^19^ of the CYP2C19*2 genotype for predicting HTPR was 37.6% (95% CI: 32.2 to 43.3%), yielding a summarized negative predictive value of only 52.3% (95% CI: 44.7% to 59.7%) and a negative likelihood ratio of only 0.77 (95% CI: 0.68 to 0.86). Thus, CYP2C19 genotyping would contribute little to excluding the risk of HTPR or MACE. Routine CYP2C19*1/*2 genotyping of all clopidogrel‐treated patients would fail to solve the problem of high on‐treatment platelet reactivity. HTPR in clopidogrel‐treated patients is indeed dependent on various other factors such as high body weight or high body mass index, clopidogrel absorption, drug‐drug interaction, underlying diseases such as diabetes, renal failure, old age, and the presence of an acute coronary syndrome.^20–22^ However, after exclusion of all identifiable genetic and non‐genetic factors, a large proportion of the variation in clopidogrel pharmacokinetics and pharmacodynamics remains unexplained at present.^23^

In recent months, physicians have been targeted by aggressive marketing from the manufacturer of the Spartan RX CYP2C19 device (Spartan Biosciences), designed for rapid identification of CYP2C19*2 carriers. This device was recently tested in the Reassessment of Antiplatelet Therapy Using an Individualized Strategy Based on Genetic Evaluation (RAPID GENE) study,^24^ which addressed the issue of tailored treatment in CYP2C19*2 carriers only, using the newer thienopyrine drug prasugrel, whose bioactivation is not significantly affected by CYP genotypes.^25^ The working hypothesis of the study was confirmed as none of the 23 CYP2C19*2 carriers allocated to prasugrel had HTPR after 7 days of treatment, compared to 7 of the 23 CYP2C19*2 carriers allocated to standard clopidogrel treatment (*P*=0.009). However, this strategy failed to identify 18 patients with HTPR (9.6% [95% CI, 5.8 to 14.8] of the total population of 187 patients) who were not CYP2C19*2 carriers. Furthermore, this false‐negative rate of 9.6% is probably underestimated. Indeed, such patients were even more numerous in the Escalating Clopidogrel by Involving a Genetic Strategy‐ Thrombolysis In Myocardial Infarction 56 (ELEVATE‐TIMI56) study^26^ (23% [95% CI, 17 to 29] of 335 enrolled PCI patients) and in the Antiplatelet Drug Resistances and Ischemic Events (ADRIE) study^10^ (39% [95% CI, 34 to 44] of 538 enrolled stable cardiovascular outpatients). Thus, a strategy tailoring anti‐P2Y12 therapy to CYP2C19*2 carrier status would ignore the 10% to 39% of clopidogrel‐treated patients who have HTPR not associated with CYP2C19*2, leaving them exposed to a 4‐ to 8‐fold higher risk of recurrent ischemic events, including death from stent thrombosis.^27–28^ Conversely, using a global phenotype‐based strategy with a low VerifyNow P2Y12 cut‐off (208 P2Y12 reaction units [PRU]), only 10/2930 patients in the Testing Platelet Reactivity In Patients Undergoing Elective Stent Placement on Clopidogrel to Guide Alternative Therapy With Prasugrel (TRIGGER‐PCI) study still had HTPR (0.3%, 95% CI [0.2 to 0.6]).^29^ Altogether, 2C19*2 genotyping is technically reliable, can now be rapidly performed, and provides an unambiguous and permanent categorization for an individual patient, but it lacks sufficient predictive capability to be used on its own. The alternate platelet reactivity approach also has its own set of limitations, including the absence of a standardized technique and universal cut‐offs, and the variability of the phenotype over time. When compared with CYP genotyping head‐to‐head, platelet function testing emerges as a better, albeit imperfect predictor of MACE.^30–31^ In a large nonrandomized prospective study, patients with HTPR remained at an increased risk of MACE despite a higher maintenance dose of clopidogrel or ticlopidine.^32^ Some prospective studies suggested that tailored anti‐P2Y12 treatment is associated with a lower risk of stent thrombosis,^33–35^ but this was not confirmed in larger randomized clinical trials.^36–37,29^ Ongoing studies of strategies incorporating genotyping, phenotyping, and clinical data in a stratified and sequential approach may give more favorable results. Alternatively, the use of new P2Y12 inhibitors such as prasugrel and the nonthienopyridine drug ticagrelor in all patients might largely overcome the problem of HTPR without the need for testing. Pending the results of additional controlled studies, we consider that “personalized” antiplatelet treatment based on CYP2C19 genotyping has no valid place in clinical practice yet, and that there is currently no urgency for CYP2C19 genotyping.
